# External validation of the international risk prediction algorithm for major depressive episode in the US general population: the PredictD-US study

**DOI:** 10.1186/s12888-016-0971-x

**Published:** 2016-07-22

**Authors:** Yeshambel T. Nigatu, Yan Liu, JianLi Wang

**Affiliations:** Department of Psychiatry, Cumming School of Medicine, University of Calgary, Calgary, Canada; Mathison Centre for Mental Health Research and Education, Hotchkiss Brain Institute, University of Calgary, Calgary, Canada; Department of Community Health Sciences, Cumming School of Medicine, University of Calgary, Calgary, Canada

**Keywords:** Depression, Prediction algorithm, Validation, Risk factors

## Abstract

**Background:**

Multivariable risk prediction algorithms are useful for making clinical decisions and for health planning. While prediction algorithms for new onset of major depression in the primary care attendees in Europe and elsewhere have been developed, the performance of these algorithms in different populations is not known. The objective of this study was to validate the PredictD algorithm for new onset of major depressive episode (MDE) in the US general population.

**Methods:**

Longitudinal study design was conducted with approximate 3-year follow-up data from a nationally representative sample of the US general population. A total of 29,621 individuals who participated in Wave 1 and 2 of the US National Epidemiologic Survey on Alcohol and Related Conditions (NESARC) and who did not have an MDE in the past year at Wave 1 were included. The PredictD algorithm was directly applied to the selected participants. MDE was assessed by the Alcohol Use Disorder and Associated Disabilities Interview Schedule, based on the DSM-IV criteria.

**Results:**

Among the participants, 8 % developed an MDE over three years. The PredictD algorithm had acceptable discriminative power (C-statistics = 0.708, 95 % CI: 0.696, 0.720), but poor calibration (*p* < 0.001) with the NESARC data. In the European primary care attendees, the algorithm had a C-statistics of 0.790 (95 % CI: 0.767, 0.813) with a perfect calibration.

**Conclusions:**

The PredictD algorithm has acceptable discrimination, but the calibration capacity was poor in the US general population despite of re-calibration. Therefore, based on the results, at current stage, the use of PredictD in the US general population for predicting individual risk of MDE is not encouraged. More independent validation research is needed.

## Background

Major depression is a prevalent mental disorder in the general population and imposes considerable burden on society [1–3]. According to the Global Burden of Disease study, major depression is a leading cause of disability at all ages worldwide [4]. By 2030, major depression is expected to rank first in disease burden in the high-income countries [2]. The average lifetime and 12-month prevalence of major depression were 14.6 % and 5.5 % in high-income income countries, respectively [5]. In the US general population, the lifetime prevalence of major depression was 16 % [3].

The prevalence of major depression is influenced by incidence and episode duration [6]. Major depression is highly recurrent in general populations and clinical settings. It is well recognized that the risk of recurrence increases with the number of previous episodes. Preventing new or incident cases of major depression can reduce the overall disease burden of major depression on society [7]. One of the challenges in the prevention of major depression is its multi-factorial etiology. In the past decades, population-based studies across the world have identified a number of risk factors for major depression, including age, sex, educational level, marital status, employment status, ethnicity, living alone or with others, physical illness, lifetime depression, stress, financial strain, self-rated physical and mental health, alcohol use, childhood adversity, major life events, poor social support and experiences of discrimination on grounds of sex, age, ethnicity, appearance, disability, or sexual orientation [8–12].

For the purpose of early identification and early intervention, health professionals and policy makers need tools that can accurately identify individuals who are at high risk of developing major depression in the future so that preventive actions can be taken. In the clinical setting, predictive risk algorithms are embedded in clinicians’ daily practice as the primary tool to estimate individuals’ risks of future disease. There have been multivariable risk prediction algorithms for first onset [8], new onset [9, 13] and recurrent [7] major depressive episode (MDE) in different populations and settings. The PredictD algorithm was developed in primary care attendees in 6 European countries, who were between the ages of 18 and 75 and who did not have MDE in the past 6 months [13]. The algorithm was developed to predict individuals’ risks of MDE in the next 12 months. Because the predictive performance of a model based on the development data is often optimistic, it is important that the developed model is validated in different populations, in different geographic regions or in different time periods [14, 15]. This addresses the accuracy of a model in individuals from a different but plausibly related population. However, most reports evaluating prediction models focus on the issue of internal validity, leaving the important issue of external validity behind. The PredictD international algorithm had good performance in the development data. It was validated with Chilean data as part of the PredictD study. To our knowledge, the algorithm has not been validated in populations besides those in the PredictD study. In the present study, the objective was to validate the PredictD algorithm in the US general population.

## Methods

### Study design and population

We used the data from the longitudinal cohort of the US National Epidemiological Survey on Alcohol and Related Conditions (NESARC). The NESARC was a nationally representative survey of the US general population funded by the National Institute on Alcohol Abuse and Alcoholism. Wave 1 of the NESARC was conducted between 2001 and 2002 and included 43 093 respondents aged 18 years and older. Wave 2 of the NESARC was conducted between 2004 and 2005, about 3 years after Wave 1. 34 653 participants of the original Wave 1 sample completed interviews at Wave 2. Of the 34,653 NESARC participants, we included 29,621 participants who were aged 18 to 75 years and who did have MDE in the past year at Wave 1, which resembled the sample of the PredictD study. A detailed description of the design and field procedures of the NESARC has described elsewhere [16, 17]. The NESARC data were collected using face-to-face computer-assisted interviews by trained lay interviewers. As current study was a secondary data analysis of public use data, ethics review was waived by the Conjoint Health Research Ethics Review Board of University of Calgary.

### Assessment of mental disorders

MDE and other Axis-I and Axis-II mental disorders were assessed using the Alcohol Use Disorder and Associated Disabilities Interview Schedule (AUDADIS), based on the DSM-IV criteria [18, 19], a fully structured diagnostic interview that can be used by trained lay interviewers. Lifetime and past-year diagnoses were assessed at Wave 1. At Wave 2, diagnoses since Wave 1 were assessed.

### Predictors

There are 10 predictors in the PredictD algorithm. The NESARC contains the following predictors similar with those in the PredictD study, which were measured using the same instruments or similar questions:

1. Age (years)
2. Sex (Male/Female)
3. Educational status was defined as completing beyond secondary, secondary/high school, primary/no education and trade/other education.
4. For the predictor “Difficulties in paid and unpaid work”, the NESARC did not include questions about work stress as measured by the Job Content Questionnaire in the PredictD. We used the answers to the questions: experiencing difficulties with boss or co-workers, and being fired or laid off in the past 12 months, as a proxy predictor. It was dichotomized as having or not having difficulties for paid or unpaid work.
5. Physical component score (PCS) measures physical quality of life in the past month, which was assessed by the Medical Outcomes study—Short Form (SF-12, version 2) [20] in both the NESARC and the PredictD study.
6. Mental component score (MCS) measures past month mental quality of life. It was assessed by Medical Outcomes study—Short Form (SF-12, version 2) [20] in both the NESARC and the PredictD. The PCS and MCS scores were standardized, ranging from 0 to 100.
7. History of depression in first-degree relatives was assessed as part of the AUDADIS [18]. Same as the PredictD study, the NESARC participants were asked about whether their biological parents and siblings ever had depression (yes/no).
8. Experience of discrimination was assessed using NESARC questions on the grounds of physical disability, race-ethnicity, gender, sexual orientation, religion and being overweight. These questions were asked at Wave 2 and accommodated two time periods: the past 12 months, and prior to the past 12 months. In this study, we assumed that people’s experience of discrimination did not have a significant change over a short period of time (e.g., 2 years). Therefore, we used participants’ answers about experience of discrimination prior to the past 12 months as an indicator for discrimination. Same as the PredictD study, the experience of discrimination was categorized into three levels: no discrimination, having discrimination in one of the above grounds/area, and in more than one area.
9. Any lifetime MDE prior to 12 months at Wave 1 (yes/no) was assessed using AUDADIS based on the DSM-IV criteria [18, 19].
10. Country: As we validated the PredictD model in the US population, in our validation, we entered “0” for the coefficient of “country”, assuming that the NESARC participants were similar with the UK sample.

### Statistical analysis

The outcome variable of the prediction algorithm was new onset of MDE ascertained with Wave 2 data. We applied the PredictD algorithm in the NESARC data, using the exact same coefficients of the nine predictors in the PredictD model: age, sex, education, experiencing difficulties at work and laid off, physical and mental health, family history, discrimination and lifetime depression (Table 1).

**Table 1:** Risk factors in the PredictD algorithm and the regression coefficients after shrinkage

Prognostic factor	Levels in factor	Coefficients^a^
Constant		1.155
Age	Each year	−0.005
Sex	Female	
	Male	−0.212
Education	Beyond secondary education	
	Secondary education	0.089
	Primary/no education	0.409
	Trade/other	0.566
Difficulties in paid and unpaid work	No difficulties or often supported	
	Difficulties without support	0.366
Physical health	Each point on SF-12 subscale score	−0.030
Mental health	Each point on SF-12 subscale score	−0.055
First-degree relative with emotional problem	No	
	Yes	0.395
Discrimination	No	
	In one area	0161
	In more than one area	0.736
Lifetime depression	No	
	Yes	0.489
Country	UK	
	Spain	0.23
	Slovenia	-0.729
	Estonia	-0.467
	The Netherlands	-0.115
	Portugal	-0.169

^a^ Regression coefficients after shrinkage (King et al. 2008) [13]

We applied the prediction model directly to the selected NESARC participants with and without re-calibration. Re-calibration is a method of adjusting an existing model to predict risk in a new setting. It involves estimating only two new parameters that are expected to produce reasonable predictions beyond the dataset used for recalibration. The logit risk score (Z) was recalibrated to predict onset of MDE by fitting a logistic model with Z as the predictor variable, i.e. the slope (a) and intercept (b) were estimated for the model logit = a + bZ [21].

We assessed the model performance by discrimination and calibration. Discrimination is the ability of a prediction model to separate those who experienced the outcome events from those who did not. We quantified discrimination by calculating the C statistic, which is identical to the area under a receiver operating characteristic (ROC) curve when the outcome is binary, also known as AUC. Calibration measures how closely the predicted outcomes agree with actual outcomes (or accuracy). For this we used the Hosmer–Lemeshow (H–L) χ2 statistics. A χ2 statistic was calculated to compare the differences between the mean predicted and the observed risks; large P-value (i.e., greater than 0.05) indicates good calibration.

We also assessed the calibration by grouping individuals into deciles of risk and visually comparing the observed and the predicted risk, so that the overall calibration, and the areas with over or under prediction could be identified. We re-calibrated the algorithm to improve the agreement between the predicted and observed risks. All analyses were performed using Stata release 13 (Stata Corp. LP, USA).

## Results

The characteristics of the participants in the PredictD study and the NESARC are presented in Table 2. The participants in the two studies resembled each other, but slightly differed in gender, race and marital status. In the NESARC sample, the lifetime and 12-month prevalence of MDE at Wave 1 were 19.7 % and 8.6 %, respectively. The 12-month prevalence of MDE in the PredictD sample was 7.7 %.

**Table 2:** Demographic characteristics of the US and European population

Characteristics	US population	European population^a^
	N (%)	N (%)
Age (year), mean (SD)	43.8 (15.2)	48.9 (15.5)
Female	16608 (56.1)	4081 (65.9)
Marital status		
Married or living together	16 532 (55.8)	4491 (72.6)
Separated or divorced	4654 (15.7)	421 (6.8)
Single	6765 (22.8)	872 (14.1)
Widowed	1670 (5.6)	383 (6.2)
House hold status		
Not living alone	22046 (74.4)	5483 (88.6)
Living alone	7575 (25.6)	707 (11.4)
Education		
Higher education	17793 (60.1)	1879 (30.4)
Secondary	10084(34.0)	2038 (32.9)
Primary/no education	1634 (5.5)	1767 (28.6)
Trade/other	110 (0.4)	451 (7.3)
Missing	0	55 (0.9)
Employment		
Employed/full time student	20993 (71.0)	3256 (52.6)
Unemployed	989 (3.3)	300 (4.8)
Unable to work	1190 (4.0)	322 (5.2)
Retired	4032(13.6)	2269 (36.7)
Missing	2417 (8.2)	43 (0.7)
Professional status		
Yes	4628 (15.6)	1313 (21.2)
Missing	4815 (16.3)	143 (2.3)
Born in country of residence		
Yes	24727 (83.5)	5655 (91.4)
Missing	80 (0.3)	87 (1.4)
Ethnicity		
White European	22466 (75.8)	5988 (96.7)
Missing	0	72 (1.2)

^a^ United Kingdom, Spain, Slovenia, Portugal, The Netherlands, Estonia

### External validation of the PredictD study in the US population

**Fig. 1:**
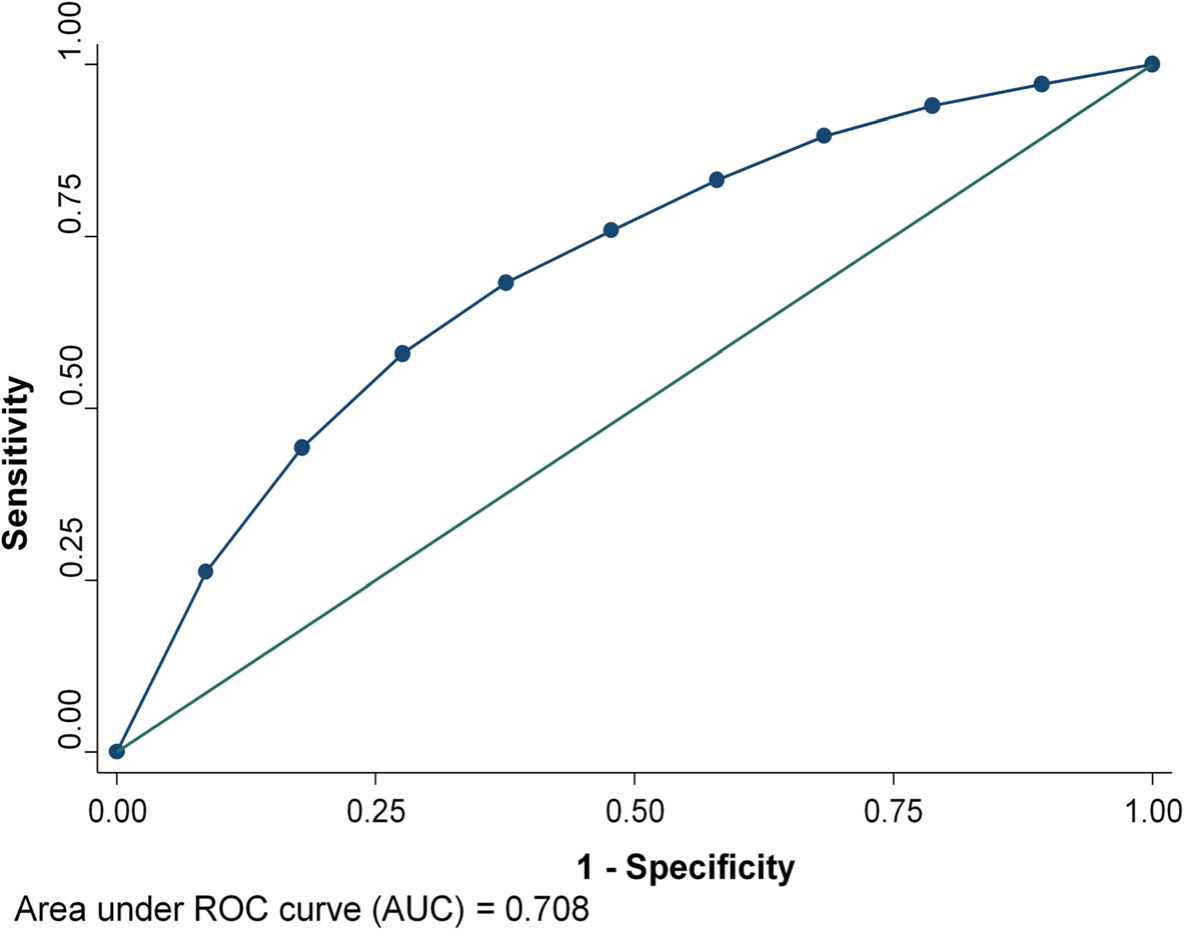
Discrimination graph: Receiver operating characteristic (ROC) curve

**Fig. 2:**
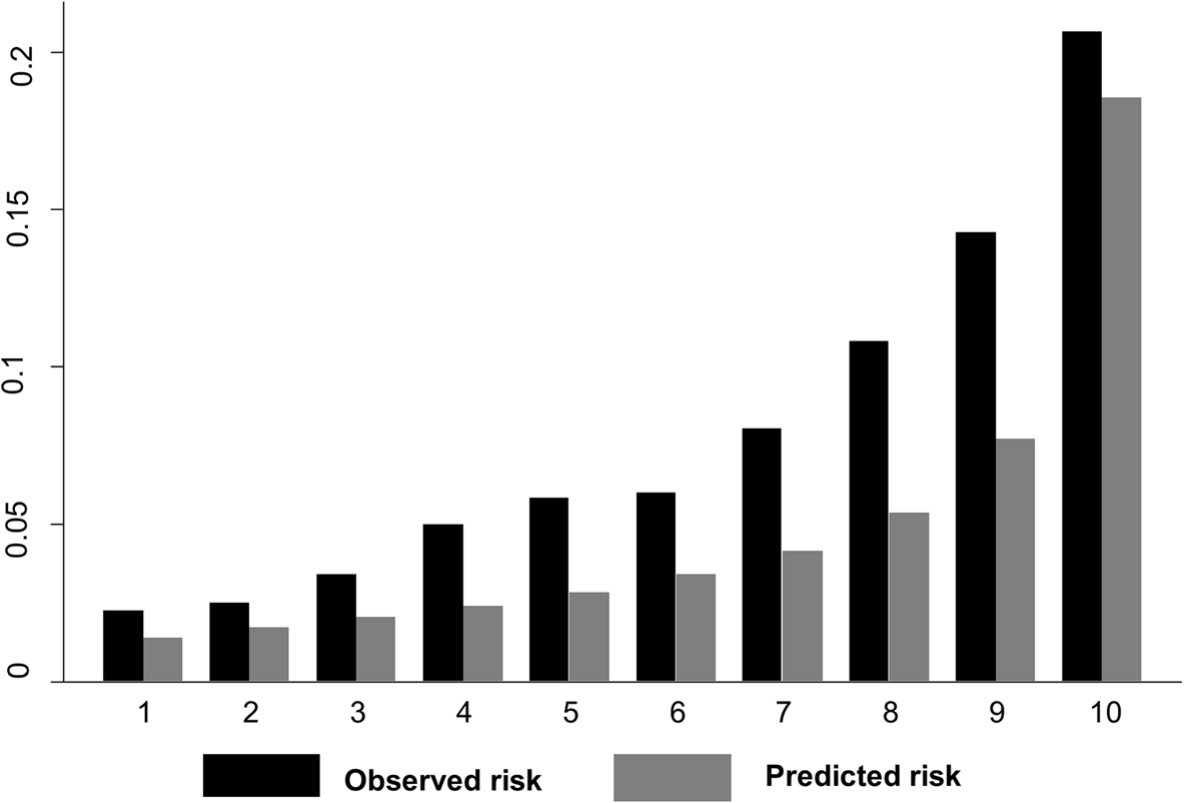
The predicted risk proportion versus observed risk proportion of major depressive episode by 10 groups

In NESARC participants who had complete data on all predictors (*n* = 24311), 8 % developed an MDE over three years. The C-statistic for the PredictD algorithm in the NESARC data was 0.708 (95 % CI: 0.696 - 0.720), and had poor calibration, as assessed by the Hosmer-Lemeshow (H-Lχ2) test with *p* < 0.001. Figure 1 shows the ROC curve; the diagonal indicates no discrimination above chance. In the PredictD participants, the algorithm had a C statistic of 0.790 (95 % CI: 0.767 - 0.813) with a perfect calibration. The NESARC data showed that, based on the PredictD algorithm, the observed and the mean predicted risk of MDE were 8 % and 5 %, respectively. As indicated in Fig. 2, the observed and predicted risks of MDE in the highest decile of risk score in the NESARC participants were 21 % and 19 %, respectively. This suggests that the PredictD model tends to under estimate the risk of MDE in the US general population, overall and in the high risk groups. Comparing the 10th (mean predicted risk = 22.7 %) and the first (mean predicted risk = 3.1 %) decile group, the PredictD model could identify over 7-fold of risk. Using the minimum risk of the 8th, 9th, and 10th decile group as cutoffs, the sensitivity (specificity) was 58.0 % (72.1 %), 44.3 % (82.1 %), and 26.2 % (91.4 %), respectively (Fig. 1). Overall, the positive and negative predicted values were 37.4 % (95 % CI: 28.6 - 47.0 %), and 92.3 % (95 % CI: 91.9 - 92.6 %), respectively.

**Fig. 3:**
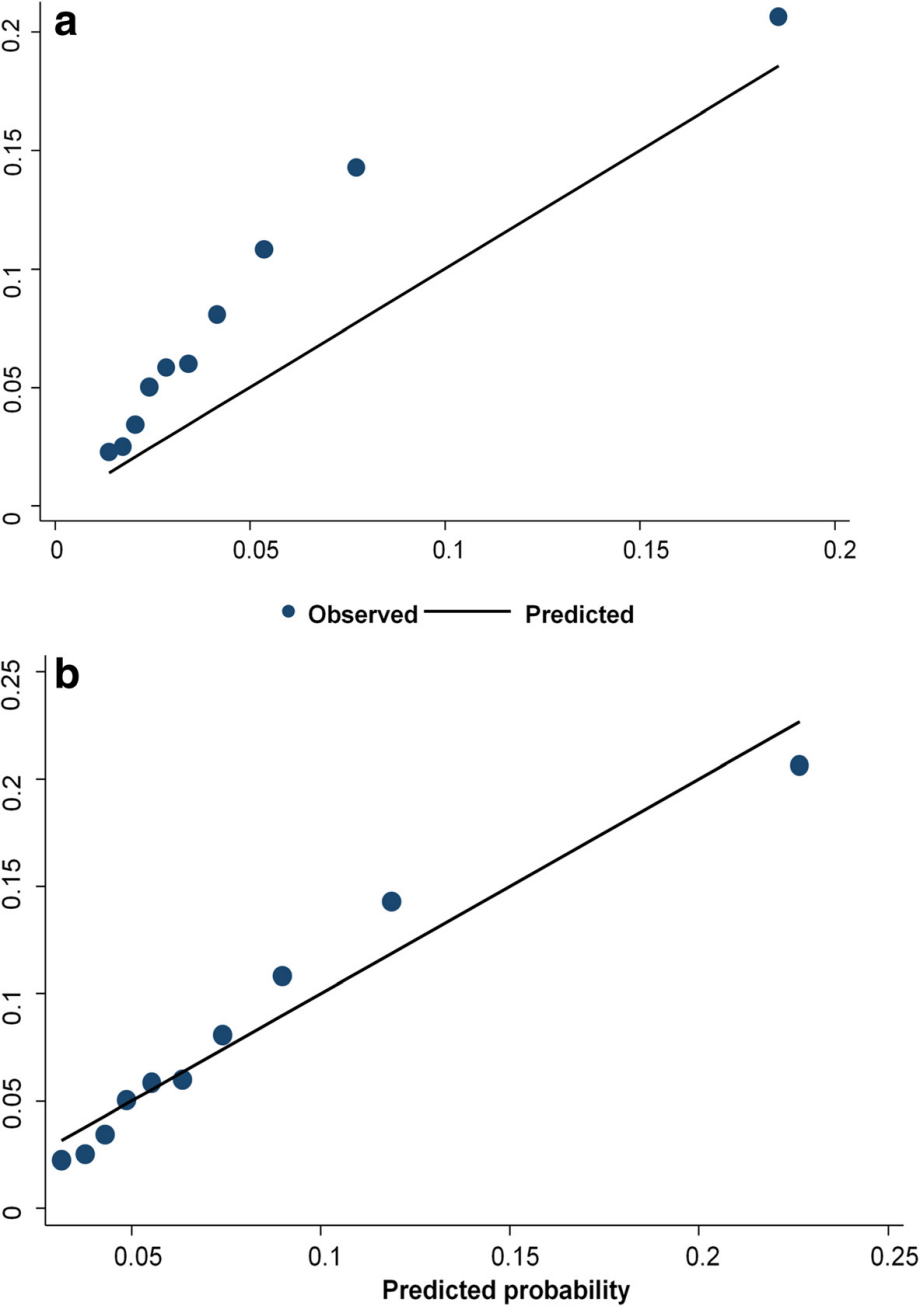
Plots of mean predicted probability against observed probability of Major Depressive. Episode within deciles of predicted risk without (**a**) and with re-calibration (**b**)

With re-calibration, the C-index score (C = 0.708) remained the same. Although the agreement between the observed and predicted risks improved with re-calibration, the goodness of fit test remained significant (H-Lχ2, *p* = 0.0001) which indicates poor calibration. In Fig. 3a and b, we plotted the mean predicted probability vs the observed probability of MDE with and without re-calibration.

## Discussion

We validated the PredictD algorithm for the new onset of MDE over three years in the US NESARC sample. The validation results showed that the PredictD algorithm had acceptable discrimination (C = 0.708) but poor calibration in the US general population. When the PredictD algorithm was applied in the NESARC, it under estimated the risk of MDE overall and in high risk groups. The PredictD was independently validated at Chilean sites as part of the PredictD study. To our knowledge, the current study was the first attempt to validate the PredictD algorithm in a different population. The absolute differences between the mean predicted and the observed risk of MDE were improved with re-calibration.

In prediction research, external validation is necessary because prediction models tend to perform better in data on which the model was developed than on new data. This difference in performance might be an indication of the optimism in the apparent performance in the derivation set. C-index provides a standardized way of comparing the discriminative power that uses different measurement units in different settings. While the distance between the predicted outcome and actual outcome (i.e., calibration) is a central to quantify overall model performance [21]. The PredictD multivariable algorithm seemed to perform reasonably well in terms of discrimination in the US general population (C = 0.708), which is consistent with the C statistic when the algorithm was applied to the Chilean data (C = 0.710) in the PredictD study [13, 22, 23]. Although the agreement between the predicted and the observed risk was improved with re-calibration, the overall calibration of the PredictD algorithm was still poor with the NESARC data. Similarly, when the PredictD algorithm was validated with the Chilean data, poor calibration was also indicated [13].

The difference in the C statistics between the PredictD study and this validation may be due to many factors. First, the PredictD algorithm was developed to predict the risk of MDE in the next 12 months, while the PredictD in NESARC was validated to predict the risk of MDE over three years. Second, the PredictD model was developed in the primary care attendees, where the incidence of MDE might be high. In the present study, we validated the PredictD in a general population sample. Third, the PredictD model included a predictor of “country” (i.e., United Kingdom (reference), Spain, Slovenia, Estonia, the Netherlands, and Portugal). To validate the algorithm, a value for “country” needs to be entered. The present validation study used the same coefficient as the UK, assuming the NESARC participants were similar with the UK sample. Fourth, we used ‘experiencing difficulties with boss or coworker and laid off’ as a proxy of ‘difficulties in paid and unpaid work’, which might partly explain the difference in C statistics. Finally, the differences in the model performance may be due to different distributions of predictors in the European and American populations.

The PredictD algorithm might perform well in the general population as much as in the primary care setting. But the calibration with the NESARC data was poor. In risk prediction research, calibration should receive more attention because it determines the model’s potential clinical utility, in combination with the model’s discriminative ability [24–27]. The validation results showed that direct application of the PredictD algorithm would under estimate the risk of MDE in the NESARC participants, leading to more false negatives. With re-calibration, the performance of the PredictD algorithm improved but was still poor. This indicated that re-calibration and/or re-estimation might be needed to achieve optimal performance prior to applying a risk prediction algorithm in a new population. Different predictors included in the prediction algorithm may also contribute to poor calibration. Wang et al’s prediction algorithm for first onset of MDE among NESARC participants had excellent calibration [8]. The model included predictors such as childhood adversities, traumatic experience, past panic attack, generalized anxiety disorder symptom, and suicidal behavior [8]. In PredictD model, these predictors were not important factors for MDE in the primary care attendees [13].

Adding other risk factors when training these models may refine risk assessment and improve the accuracy of the PredictD model in the general population. Furthermore, the development of sex-specific prediction algorithms for MDE might be important as the predictors for the risk of MDE and their predicted values may differ by sex [9].

The strength of this study is that the NESARC data were population-based and the sample size was large. To our knowledge, this is the first time that the PredictD algorithm was validated in a general population sample outside of Europe. This study also had limitations, including the fact that the NESARC relied on self-report. So reporting and recalling biases were possible. Such biases may also contribute to the inconsistencies in the predictive power of some factors in different populations. However, the instruments used in the NESARC have been validated and standardized as those in the PredictD study.

## Conclusions

The PredictD algorithm has acceptable discrimination, but the calibration capacity was poor in the US general population. Despite of re-calibration, the PredictD algorithm under estimated the risk of MDE in the NESARC sample. Therefore, based on the results, at current stage, the use of PredictD in the US general population is not encouraged. In psychiatry, there have been many attempts in developing risk prediction algorithms. However, the developed tools need to be independently validated in different populations to ensure the generalizability of the models. More independent validation research is needed.

## Abbreviations

MDE, major depressive episode; NESARC: National Epidemiological Survey on Alcohol and Related Conditions; AUDADIS: alcohol use disorder and associated disabilities interview schedule; DSM-IV, diagnostic statistical manual of mental disorders-4th edition; PCS, physical component score; MCS, mental component score; SF-12, medical outcomes study—short form; H-L χ2, Hosmer-Lemeshow chi-square test
